# Theoretical Investigation of Vapor Transport Mechanism Using Tubular Membrane Distillation Module

**DOI:** 10.3390/membranes11080560

**Published:** 2021-07-24

**Authors:** Adnan Alhathal Alanezi, Mohamed Bassyouni, Shereen M. S. Abdel-Hamid, Hassn Safi Ahmed, Mohamed Helmy Abdel-Aziz, Mohamed Shafick Zoromba, Yasser Elhenawy

**Affiliations:** 1 Department of Chemical Engineering Technology, College of Technological Studies, The Public Authority for Applied Education and Training (PAAET), Shuwaikh 70654, Kuwait; 2 Department of Chemical Engineering, Faculty of Engineering, Port Said University, Port Said 42526, Egypt; migb2000@gmail.com; 3 Materials Science Program, University of Science and Technolog, Zewail City of Science and Technology, October Gardens, 6th of October City 12578, Egypt; 4 Department of Chemical Engineering, Egyptian Academy for Engineering and Advanced Technology Affiliated to Ministry of Military Production, Al Salam City 3056, Egypt; Shereenahmed@eaeat.edu.eg; 5 Civil Engineering Department, Faculty of Engineering, South Valley University, Qena 83521, Egypt; hsahmad@kau.edu.sa; 6 Chemical and Materials Engineering Department, King Abdulaziz University, Rabigh 21911, Saudi Arabia; mzoromba@kau.edu.sa; 7 Chemical Engineering Department, Faculty of Engineering, Alexandria University, Alexandria 21544, Egypt; 8 Chemistry Department, Faculty of Science, Port-Said University, Port-Said 42521, Egypt; 9 Mechanical Power Engineering Department, Port-Said University, Port-Said 42521, Egypt; dr_yasser@eng.psu

**Keywords:** desalination, membrane distillation, tubular membrane, energy, distilled water flux

## Abstract

This paper’s primary objective is to examine the vapor delivery mechanism through a tubular membrane distillation (MD) module. Experiments were conducted utilizing a hydrophobic tubular membrane module with a pore size of 0.2 µm. To establish the mass transport mechanism of water vapor, tests were carried out first with pure water as a feed. The permeate flow was then determined using NaCl aqueous feed solutions. Distilled water flux at diverse feed temperatures, feed flow rates, and feed salt concentrations was investigated. The permeate flux improved linearly with rising temperature and flow rate of the feed, however, it declined with feed concentration. Increasing temperature from 40 to 70 °C increased the permeate flux by a factor of 2.2, while increasing the feed flow rate from 60 to 120 L/h increased the permeate flux by a factor ranging from 0.7 to 1.1 depending on feed temperature. Using the Dusty gas model (DGM) the mass transport of water vapor is estimated in the membrane pores. The results showed that the water vapor delivery is controlled by way of the Knudsen molecular diffusion transition mechanism and its version changed into one capable of predicting the permeate fluxes. The mass transfer coefficient calculated and located using the Knudsen molecular transition version agreed properly with the corresponding experimental value. The delivery resistances were affected by working parameters, along with feed temperature, flow rate, and concentration. The mass transfer resistance of the membrane became the predominant controlling step to the MD process.

## 1. Introduction

Membrane distillation (MD) is a low-temperature membrane purification technique that separates vapor from a liquid solution by flowing it through membrane pores. The temperature differential between feed and permeate generates driving force across the hydrophobic membrane. The vapor–liquid equilibrium is used to guide the MD separation process [1,2,3]. Membrane distillation is useful since it is inexpensive and widely available. This characteristic conserves energy in comparison to traditional desalination procedures [4,5]. Most academics believe this is a far superior alternative to typical desalination methods such as reverse osmosis (RO), multi-stage flash distillation (MSF), electro-dialysis (ED), and so on. MD generates ultrapure water without using excessive heat or pressures [1,5,6,7,8]. Energy efficiency is one of today’s most pressing issues. The primary concern is energy demand, energy policy, pollution, and economy. Using waste heat from other operations can improve energy efficiency and save operating costs. One option is to utilize sorption chillers to generate cooled and desalinated water [9].

This work aims to theoretically investigate the mechanism of the water vapor transport mechanism in membrane distillation. Different models will be tried and compared with the experimental results.

The study will make it possible to estimate the permeate flux under different operating conditions depending on the convergence between the suggested theoretical model and obtained experimental results. This will save much time and effort in the design of a membrane distillation unit.

### 1.1. Transport Process

**Figure 1:**
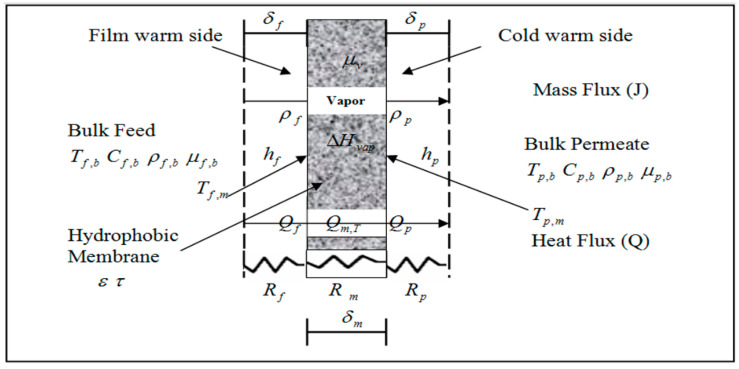
Heat and mass transfer profiles in membrane distillation.

Membrane distillation (MD) is a technique that includes both heat and mass transport. In MD, water vapor particles move from the heated feed to the condensing borders [10,11,12,13]. Figure 1 depicts transport patterns in MD because each process requires heat and mass transfer characteristics.

### 1.2. Heat Transfer

Water vapor transportation in membrane distillation may be a concurrent heat and mass transference process where the heat transfer within the MD system is often summarized in 3 steps as shown in Figure 1.

(I) Heat convection is found at the membrane surface from the bulk input to the vapor-liquid.
(II) Evaporation and conductivity through the micro-porous membrane.
(III) Heat convection from the vapor/liquid boundary to the bulk permeate at the membrane face (i.e., the thermal physical phenomenon of the permeate side) [14,15].

The evaporation through the membrane cools the feed and also the resultant gradient across the membrane translates into a lower vapor pressure gradient, successively leading to the reduction of the driving force [16,17].

## 2. Methods

### 2.1. Heat Transfer across the Membrane

The total heat flux of the feed solution and infiltrate bulk fluids (Q) is obtained by the addition of the two heat fluxes occurring in the hydrophobic membrane, which are the latent heat of vaporization $\left(Q_{v}\right)$ and the conduction $\left(Q_{c}\right)$ through the vapor contained inside the membrane holes and the membrane material. The water component vaporizes at the membrane’s exterior on the feed side, is emitted from the membrane pores, and condenses on the vapor–liquid boundary at the permeate lateral with heat flux written as:

(1) $$Q_{v}=J\cdot \Delta H_{v}$$

where $Q_{v}\left(W/m^{2}\right)$ is a form of energy transferred to the liquid in the vapor stream in the manner of latent heat to provide the needed heat of vaporization, and to compensate for that heat by condensation on the other lateral plane of the membrane, $J\left(\mathrm{kg}/m^{2}s\right)$ is the water flux through the membrane and $\Delta H_{v}\left(\mathrm{kJ}/\mathrm{kg}\right)$ is the latent heat of vaporization of water vapor.

The second heat flux by conduction is due to the temperature variations between the two sides of the membranes and is given by:

(2) $$Q_{c}=h_{m}\left(T_{fm}-T_{pm}\right)$$

where $T_{fm}$ and $T_{pm}$ are membrane surface temperatures at feed solution and permeate side, respectively, and $h_{m}\;\left(W/m^{2}K\right)$ is the heat transfer coefficient of the membrane and is defined as

(3) $$h_{m}=\frac{k_{m}}{\delta_{m}}$$

where $\delta_{m}\left(m\right)$ is membrane thickness and $k_{m}\left(W\cdot m^{-1}\cdot K^{-1}\right)$ is the average heat conductivity of membrane material and the vapor in the membrane pores:

(4) $$k_{m}=\varepsilon \cdot k_{PA}+\left(1-\varepsilon \right)k_{g}$$

where ε is the porosity of membrane, and $k_{PA}$ and $k_{g}\left(W/m\cdot K\right)$ are the thermal conductivity of the membrane sheet, and the water vapor/air mixture in the membrane pores, respectively. It is reasonable to take the water vapor/air mixture as one gas in the membrane pores because from the table below (Table 1), it is obvious that there is just a small difference in water vapor and air thermal conductivities [18].

The total heat transfer (flux) through the membrane can be obtained by combining the two heat fluxes occurring in the system by vaporization and conduction. By combining Equations (1) and (2), the total heat transfer is given by Equation (5):

(5) $$Q=Q_{v}+Q_{c}=J\cdot \Delta H_{v}+\frac{k_{m}}{\delta_{m}}\left(T_{fm}-T_{pm}\right)$$

At equilibrium, the total heat flux is equal to heat flux through the polarization layers. By assuming that the liquid thermal conductivities and the boundary layer thickness on each side of the membrane are the same, the heat flux can rewrite as:

(6) $$Q=\frac{k}{\delta }\left(T_{f,b}-T_{fm}\right)=\frac{k}{\delta }\left(T_{pm}-T_{p,b}\right)$$

where δ and k are the thickness and thermal conductivity at the polarization layers, respectively.

By manipulating Equations (5) and (6), the interface temperatures at the membrane can be evaluated, so that the vapor pressures at the membrane sheet can also be calculated using the Antoine equation, after which an iterative approach can be used to compute the mass flux (*J*) using Equations (31) and (32) [19].

(7) $$T_{fm}=\frac{\left(T_{fb}\left(\frac{k}{\delta }+\frac{k_{m}}{\delta_{m}}\right)+\frac{k_{m}}{\delta_{m}}T_{pb}-J\cdot \Delta H_{v}\right)}{\left(2\frac{k_{m}}{\delta_{m}}+\frac{k}{\delta }\right)}$$

From Equation (6), the temperatures of membrane surface at permeate side can be calculated as follow:

(8) $$T_{pm}=T_{fb}-T_{fm}+T_{pb}$$

#### 2.1.1. Heat Transfer Mechanism Along with Boundary Layers

In MD, the heat transfer through feed and permeate boundary layers influences the mass transfer rate and depends on the properties of streams and the hydrodynamic conditions. The heat flux $Q_{f}\left({\mathrm{Wm}}^{-2}\right)$ depends on the thermal boundary layer $h_{f}\left(W\cdot m^{-2}\cdot K\right)$ and the temperature difference across the feed side and feed membrane interface. It can be written as:

(9) $$Q_{f}=h_{f}\left(T_{f}-T_{fm}\right)$$

Heat flux within the thermal boundary layer at the permeate side can be obtained in a similar manner as at the feed side,

(10) $$Q_{p}=h_{p}\left(T_{pm}-T_{p}\right)$$

where $h_{p}\left(W\cdot m^{-2}\cdot K^{-1}\right)$ is the coefficient of film heat transfer at the bulk permeate in the thermal boundary layer [14]. However, the feed and permeate boundary layer heat transfer coefficients can be obtained using the following empirical correlation equation:

(11) $$Nu=a_{1}Re^{a_{2}}Pr^{a_{3}}$$

where $Nu=\frac{h\;d}{k^{T}}$, $Re=\frac{\rho \;V\;d}{\mu }$, $Pr=\frac{\mu C_{p}}{k^{T}}$.

where Nusselt number (*Nu*), Prandtl number (*Pr*), Reynolds number (*Re*), h is the heat transfer coefficient, $k_{L}$ is the liquid thermal conductivity, *d* is the diameter of the membrane, ρ is the fluid density, ν is the fluid velocity, *µ* is the fluid viscosity. Many empirical correlation equations can be found in the literature for evaluating boundary layer heat transfer coefficients.

For laminar flow occurring within a circular tube (tubular conducts) [20]:

(12) $$Nu=0.13\;Re^{0.64}\;Pr^{0.38}$$

For turbulent flow occurring within a circular tube (tubular conducts) [21]:

(13) $$Nu=0.023\;Re^{0.33}{\left(\frac{u}{u_{w}}\right)}^{0.14}$$

where $u_{w}$ is the liquid velocity at the membrane surface. The heating/cooling correction factor ${\left(u/u_{w}\right)}^{0.14}$ is always neglected in the MD process.

#### 2.1.2. Temperature Polarization Coefficient

The primary resistances are often located within the membrane’s border layer as well as on each side of the membrane. Temperature polarization coefficient (TPC) and concentration polarization (CP) can be used to simulate boundary layer resistance. TPC indicates heat transfer boundary layer resistance, which relates to overall heat transfer resistance [20,21]:

(14) $$\mathrm{TPC}=\frac{\Delta T}{\Delta T_{max}\frac{\left(T_{fm}-T_{pm}\right)}{\left(T_{fb}-T_{pb}\right)}}$$

The temperature polarization coefficient (TPC) is an indication of the efficiency of the MD process. The TPC drop is mainly in the range of 0.4 to 0.8. It approaches unity for the well-proposed system when the process is limited by mass transfer. In a poorly designed system, the TPC approaches zero, which is attributed to the high heat resistance of boundary layers. Thus, this process is limited by heat transfer [19,22].

### 2.2. Mass Transfer

The mass transfer of water in the membrane distillation process occurs in two steps: (i) the first occurs at the bulk feed via the boundary layer, and (ii) the second action occurs across the membrane itself. Materials diffusion controls mass transport across the membrane, resulting in a concentration gradient [19,23].

#### 2.2.1. Mass Transfer across the Membrane

MD modeling may be done in two ways. The first is interested in simulating the transport mechanism via the hydrophobic membrane. The second focus is in using anywhere modeling to estimate permeate flux under given operating parameters [24]. The usual formulations give a linear connection between the mass flux (*J*) and the water vapor pressure fluctuation $\Delta P_{v}$ across the membrane to explain the water vapor transport in MD and the basic equation [25,26]:

(15) $$J=K_{m}\Delta P_{v}=K_{m}\left(P_{v1}-P_{v2}\right)$$

where $\left(K_{m}\right)$ is the membrane mass transfer coefficient or permeability, which can be a function of pressure, temperature, and the composition inside the membrane, as well as the membrane structure [porosity (ε), thickness $\left(\delta_{m}\right)$, pore size diameter $\left(d_{p}\right)$]. $\left(K_{m}\right)$ can be calculated experimentally or theoretically (Knudsen diffusion, molecular diffusion, or Poiseuille viscous flow) [27,28].

#### 2.2.2. Mass Transfer within Membrane Pores

**Figure 2:**
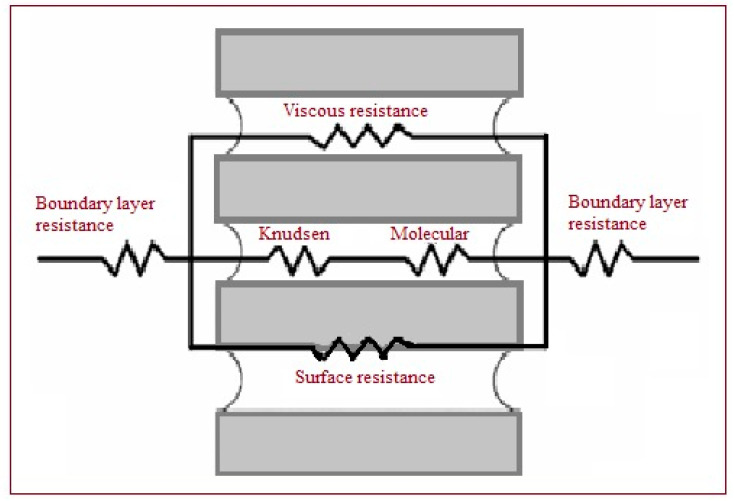
Mass transfer resistances in MD.

As depicted, by the circuit shown in Figure 2, three mechanisms regulate the mass transfer across the membrane (excluded surface diffusion) [29]:

- Knudsen diffusion (molecules–wall collision).
- Molecular diffusion (molecules–molecules collision).
- Poiseuille flow (the gas viscosity).

In this investigation, the total pressure gradient is 0, i.e., no resistance caused by gas within membrane pores. As a result, the Poiseuille viscous flow can be ignored, and surface diffusion is always ignored in MD [30,31]. As a result, the Knudsen diffusion, molecular diffusion, and Knudsen molecular diffusion transition models may be used to calculate water vapor movement through membrane pores. For the Knudsen diffusion, molecular diffusion, and Knudsen molecular diffusion models, the mass transfer flow may be expressed as:

Knudsen diffusion model:

(16) $$J_{K}=\frac{4}{3}\frac{\varepsilon d}{\tau \delta_{m}}\sqrt{\frac{M}{2\pi RT_{m}}}\Delta P_{v}$$

Molecular diffusion model:

(17) $$J_{M}=\frac{\varepsilon D_{wa}}{\tau \delta_{m}}\frac{PM_{w}}{RT_{m}}ln\left(\frac{P-P_{v1}}{P-P_{v2}}\right)$$

Knudsen molecular diffusion transition model:

(18) $$J_{K-M}=\frac{\varepsilon M_{w}}{\tau \delta_{m}}\frac{PD_{wa}}{RT_{m}}ln\left(\frac{\frac{P-P_{v2}}{PD_{wa}}+\frac{3}{4d_{p}}{\left(\frac{2\pi M_{w}}{RT_{m}}\right)}^{1/2}}{\frac{P-P_{v1}}{PD_{wa}}+\frac{3}{4d_{p}}{\left(\frac{2\pi M_{w}}{RT_{m}}\right)}^{1/2}}\right)$$

where $D_{wa}\left(m^{2}/s\right)$ can be obtained from the empirical equation [22]:

(19) $$PD_{wa}=4.46\times 10^{-6}T^{2.334}$$

where the unit of $PD_{wa}\;\mathrm{is}\;\mathrm{Pa}\cdot m^{2}\cdot s^{-1}$.

#### 2.2.3. Mass Transfer through the Boundary Layers (Concentration Polarization)

**Figure 3:**
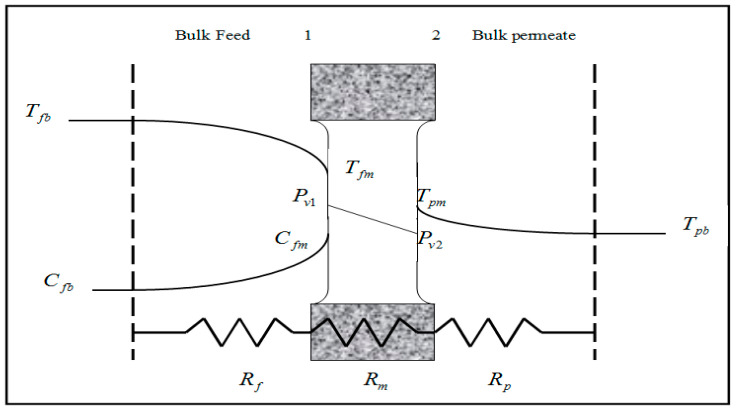
Concentration and temperature profiles in MD.

The first parts of the experiments were performed with pure water because the resistances of the boundary layer to mass transfer can be neglected. Then, the second part of the experiments was carried out using various concentrations of NaCl. The attention of concentration polarization (CP) should be raised because the boundary layers increase the total resistance to mass transfer and also the sufficient concentration of solute could cause spontaneous wetting of the membrane. From Figure 3, the boundary layers are formed (i.e., concentration polarization occurs) because of the difference in salt concentration between bulk and membrane surface sides.

Assuming the salt is completely retained by membrane and according to the mass balance across the feed solutions side boundary layers, a relationship between mass flux, *J*, the salt mass transfer coefficient $K_{s}$, and salt concentration at feed bulk $C_{fb}$ and at the membrane surface $C_{fm}$ is given by the film model [19,27]:

(20) $$C_{fm}=C_{fb}exp\left(\frac{J}{\rho K_{s}}\right)$$

where ρ is the density and the salt mass transfer coefficient is $K_{s}$ could be appraised by applying the Dittus–Boelter correlation:

(21) $$K_{s}=0.023\left({Re}^{0.8}\cdot Sc^{0.33}\right)\frac{D_{wa}}{d_{h}}$$

where *Re* is the Reynolds number, *Sc* is the Schmidt number, $D_{wa}$ is the diffusion coefficient of water vapor through stagnant air and $d_{h}$ is the hydraulic diameter (m).

Because of the salt concentration, the flux reduction is expected; consequently, the vapor pressure of water will decrease as well and can be estimated using Raoult’s law:

(22) $$P_{v}{}^{*}=\left(1-C_{fm}\right)\cdot P_{v}$$

where $P_{v}$, is the vapor pressure of pure water, $P_{v}{}^{*}$ is the vapor pressure of saltwater, and $C_{fm}$ is the mole fraction of the salt at the membrane interface.

The CPC can be given as:

(23) $$\mathrm{CPC}=\frac{C_{fm}}{C_{fb}}$$

For calculating the mass transfer coefficient of liquid in the boundary layer:

(24) $$Sh=b_{1}Re^{b_{2}}Sc^{b_{3}}$$

where

$$Sh=\frac{kd_{h}}{D_{AB}};\;Re=\frac{\nu d_{h}\rho }{\mu };\;Sc=\frac{\mu }{\rho D_{w}}$$

where $b_{1}$,$b_{2}$ and $b_{3}$ are constants, k is the liquid mass transfer coefficient; $k=D_{w}/\delta$, $D_{w}$ is the water diffusion coefficient in the liquid, μ is the bulk liquid viscosity, ν is the liquid velocity and $d_{h}$ is the hydraulic diameter.

#### 2.2.4. Transport Resistances

The total resistance in the membrane distillation process is composed of the resistances of the feed, membrane, and permeate boundary layers respectively without the presence of fouling layer (i.e., pure water).

Moreover, the mass flux can be estimated by calculating the overall mass transfer coefficient $\left(K_{ov}\right)$ as follows [32,33]:

(25) $$J=K_{ov}\Delta P_{w,b}$$

where, $\Delta P_{w,b}$ is the water vapor pressure difference at the bulk feed and bulk permeate sides, respectively.

Overall mass transfer coefficient is given below:

(26) $$K_{ov}={\left(\frac{1}{K_{f}}+\frac{1}{K_{m}}+\frac{1}{K_{p}}\right)}^{-1}$$

(27) $$K_{ov}={\left(R_{f}+R_{m}+R_{p}\right)}^{-1}$$

where, $R_{f},\;R_{m},\;R_{p}$ are the resistances at feed, membrane, and permeate boundary layers respectively. The resistances can be evaluated as follows [34]:

(28) $$R_{f}=\frac{\left(P_{f}-P_{v1}\right)}{J}$$

(29) $$R_{m}=\frac{\left(P_{v1}-P_{v2}\right)}{J}$$

(30) $$R_{p}=\frac{\left(P_{v2}-P_{p}\right)}{J}$$

For pure water, the water vapor pressure at the water–vapor interface can be calculated using the Antoine equation [19,26]:

(31) $$P_{v}=exp\left(23.1964-\frac{3816.44}{T-46.13}\right)$$

They are calculated as a function of local temperature and salt concentration using the modified Antoine equation [35]

(32) $$P_{v}^{*}=\frac{exp\left(23.1964-\frac{3816.44}{T-46.13}\right)}{1+0.57357\left(\frac{K_{ov}}{1000-K_{ov}}\right)}$$

#### 2.2.5. DCMD Thermal Efficiency

The thermal efficiency (η) of the process is defined as the ratio of the amount of heat evaporation to the total heat flux of Equation (5). Therefore, η can be expressed as [36]:

(33) $$\eta =J\cdot \Delta H_{v}/Q$$

### 2.3. Pure and Saltwater Physical Properties

The end-use models are listed below for pure water and solution characteristics of the components required by the various process models. Table 2 shows how the physical properties of water at various temperature-dependent parameters or concentration-dependent features of pure water and saline water are connected by curve fitting [37,38,39,40].

#### Numerical Model

During a one-dimensional module, simulations were carried out. The hydrophobic membrane module used in this study consisted of nine polymeric tubular membrane tubes arranged in a zigzag pattern. The module’s overall effective area was 0.1144 m^2^. The channel length in the experimental research [3,6] used to validate the mathematical model was 2.8 m. Actual modules used in industrial applications can be used for considerably longer (order of magnitude longer). Because the temperature of the feed solution decreases due to vaporization and conductive energy loss, the temperature of the permeate fluid increases due to energy gain, the flow performance might gradually decline in the stream-wise direction. The membrane structural characteristics and operating conditions used in the simulations are listed in Table 3. The membrane utilized in this study was a full-life PTFE hollow membrane with a permeability of 0.35 × 10^−4^ kg/m^2^ h Pa S at the reference temperature and pressure. As seen in Equation (27), the porosity is affected by the feed and therefore the permeate temperature on the membrane surface. The simulations were carried out at flow rates corresponding to *Ref* of 2500 and 15,000 for the feed channel. The Reynolds number for each stream is computed as *Re_n_* = (*U_n_,_ave_*, *ρ_n_*, *d_n_*, *h*)/*μ_n_*, where *U_n_,_ave_* is the average speed at the inlet, and *n* = *f*/*p* is the feed and permeate stream characteristics, respectively. The hydraulic diameter is decided from *d_n_*, *h* = 4 *A*/*P*. The inlet feed concentration, 5000 ppm, and 35,000 ppm represent brackish water and seawater desalination respectively. The feed and permeate inlet temperatures were varied feed temperature from 40 to 70 °C and 23 °C, respectively. Generally, pure and saline water fluxes, at various temperatures, flow rates, and concentrations for the feed stream have been studied.

Specifications and values of uncertainties of the measuring devices are displayed in Table 4.

## 3. Results and Discussion

### 3.1. Mechanism of Mass Transport

#### 3.1.1. The Approximated Method for Predicting Permeates Flux Using Average Temperatures of the Inlet, Outlet Membrane Module, and Relative Humidity

In this part, the average of inlet and outlet temperatures through the tubular membrane module is used to calculate the liquid feed bulk temperature $T_{fb}$, and then, by using the Antoine equation, we can estimate the vapor pressure $P_{v1}\left(T_{fb}\right)$ at bulk feed side. Since the membrane module is opened to the atmosphere, the relative humidity can be used to estimate the vapor pressure $P_{v2}\left(T_{pb}\right)$ at the bulk permeate side. Then, from the vapor pressure difference for the feed and permeate sides we can theoretically predict the permeate fluxes and mass transfer coefficients for the three diffusion mechanisms and compare them with the experimental values.

For water, the mean free path (λ) could be calculated as following [41]:

(34) $$\lambda =\frac{3\mu_{v}}{P}\sqrt{\frac{\pi RT_{m}}{8M_{w}}}$$

The vapor and liquid are assumed to be in an equilibrium state at the mean temperature and the pressure inside membrane pores. Therefore, for pure water, the vapor pressure of water is up to the saturation vapor pressure and might be calculated by using the Antoine equation, where the vapor pressure at the permeate facet will be calculated using the relative humidity (*RH*) or water activity (*a_w_*):

where Relative Humidity $\left(RH\right)=\frac{P_{v2}}{P_{v1}}$ [41,42] (λ=0.0713 μm=71.3 nm.)

The value of the mean free path of most gases is in the range of 40 and 200 nm [42]

Knudsen number $\left(K_{n}\right)$ can be used because of the initial criteria for determining the predominant mechanism for water transport through the tubular membrane module.

(35) $$K_{n}=\frac{mean\;free\;patt\;of\;water\;vapor}{membrane\;pore\;size}$$

(36) $$K_{n}=\frac{\lambda }{d_{p}}=\frac{0.0713\;\mu m}{0.72\;\mu m}=0.099$$

Since $0.01<K_{n}<1$ then, the Knudsen molecular transition diffusion mechanism regulates the mass transfer within the membrane pores. Now we can confirm the above result of $K_{n}$ from the above theoretical models, which can be used to describe water vapor transport within membrane pores. The effect of the Knudsen number on a mass transfer through a porous medium is displayed in Table 5.

**Figure 4:**
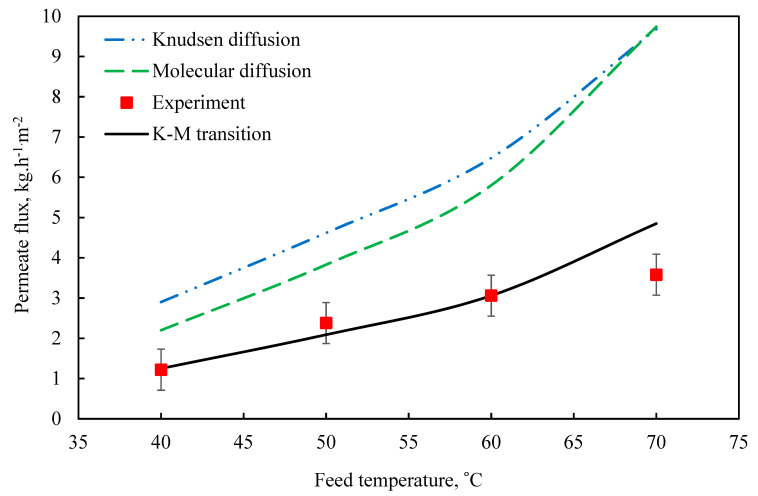
Pure water flux versus different feed temperature at *m_f_* = 60 L/h and *P* = 1 bar (K_m_ = 0.39 $\mathrm{kg}/m^{2}\cdot h\cdot \mathrm{Pa}$.

Figure 4 shows that the comparison between theoretical and experimental data [3] at feed and permeate temperature. The theoretical and experimental values of the mass transfer coefficient are summarized in Table 6.

#### 3.1.2. The Exact Method for Predicting Permeates Fluxes Using Membrane Interface Temperatures on the Feed and Permeate Side

**Figure 5:**
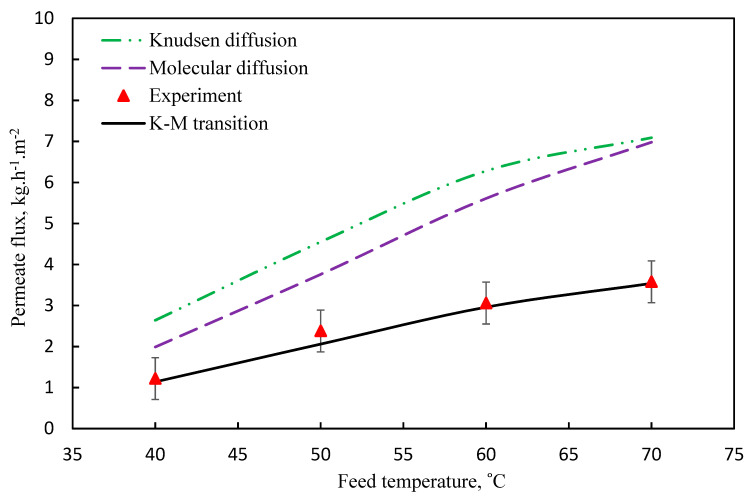
Pure water flux versus feed temperature at *m_f_* = 60 L/h and *P* = 1 bar (K_m_ = 0.38 $\mathrm{kg}/m^{2}\cdot h\cdot \mathrm{Pa}$).

In this part, membrane interface temperatures are calculated using the heat and mass transfer equations at operating conditions and membrane characteristics. Using the Antoine equation, the vapor pressure difference for each side of the membrane can be calculated, and then the theoretical permeate fluxes and mass transfer coefficients can be calculated and compare with the experimental values. The effect of temperature on the mass transfer resistance is displayed in Table 7. Theoretical and experimental fluxes for membrane interface temperatures are shown in Figure 5.

### 3.2. Model Validation

**Figure 6:**
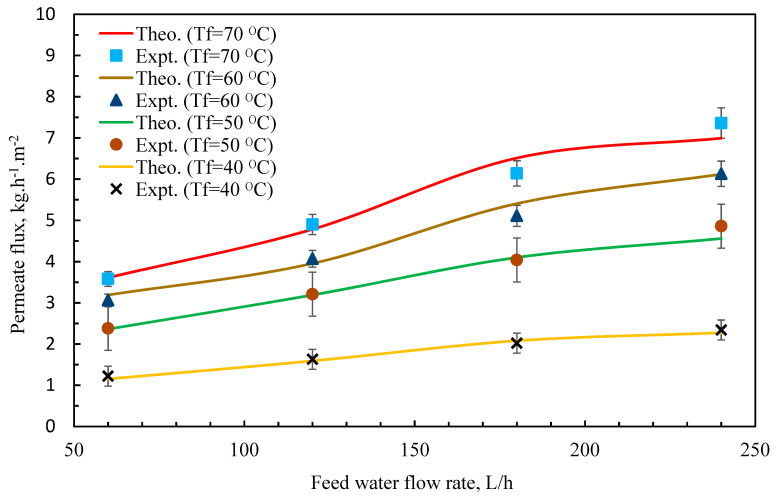
Comparison between theoretical and experimental flux at various feed water flow rate and temperature.

The conclusions of the mathematical model were then validated against the entirely different experimental results. Figure 6 compares the anticipated mass fluxes and hence the measured vapor fluxes over a range of deionized feedwater temperatures (40–70 °C). The model predicted that the DCMD flow will behave exponentially as a function of feed water temperature. Such behavior is not only confirmed by our experimental findings, but it is also suggested in published AGMD literature [40,43,44].

However, the validity of the mathematical model should not be assessed just on how well it forecasts the trend of the process. It should also be rated on how well it predicts the experimental outcome. The present goal of building this model is to use it as a tool for assessing the DCMD technique and scaling it up. As a criterion for determining the validity of our module, such a goal may need to be loosened. Nonetheless, the model’s prediction was within the experimental error range. To validate the model, we tend to replace the deionized water (feed) with saltwater to see how well the model forecasts the vapor flux at 35,000 ppm seawater salinity. The distillate physical phenomenon was constantly monitored to ascertain any pore wetting which will ensue and therefore the distillate conductivity was invariably below 20 μS.

Regression analysis agreed with experimental data fitting using a quadratic polynomial model with coefficients of determination (R^2^) values of 0.986, 0.992, and 0.988 for permeate flux, feed temperature, and feedwater flow rate, respectively.

### 3.3. Effect of Feedwater Flow Rate and Salt Concentration on Permeate Flux

**Figure 7:**
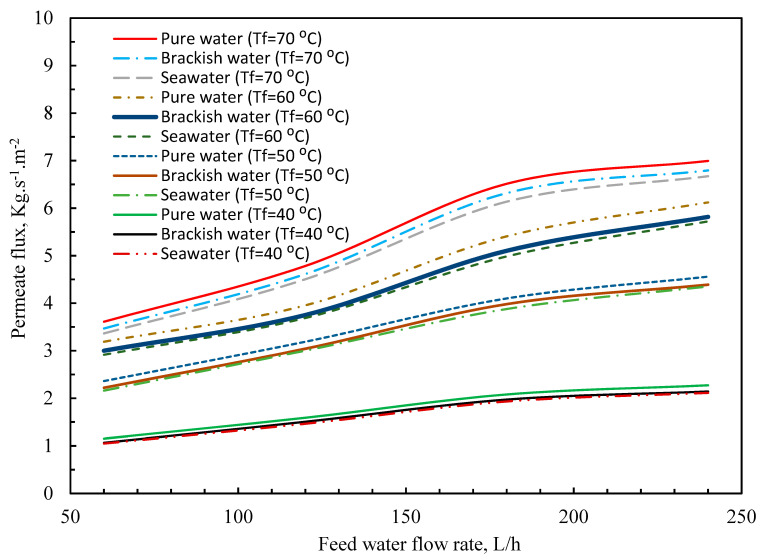
Pure, brackish, and seawater water flux versus feed temperature and water flow rate.

**Figure 8:**
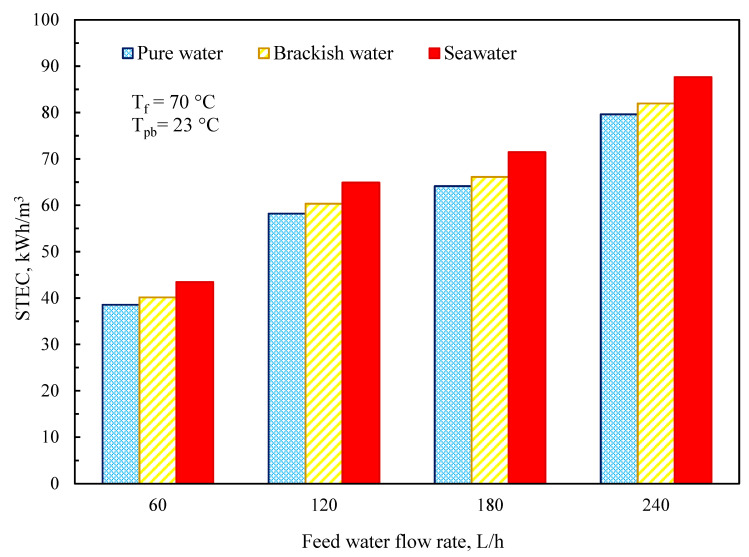
Feed Reynolds number versus feedwater flow rate (*T_fb_* = 70 °C, *T_pb_* = 23 °C).

The impact of the feedwater flow rate, as illustrated in Figure 7, was to enhance the permeate flux [45,46]. This pattern might be explained by the fact that an increase in mf junction rectifier leads to an increase in heat transfer coefficients. The temperatures of the boundary layer, *T_mf_* and *T_mp_*, grew closer to the temperatures of bulk solutions, *T_bf_* and *T_bp_*, as the coefficient’s value increased. This resulted in a higher temperature distinction and, as a result, an increase in permeate flow (*J*) [3,47,48,49]. Figure 8 indicates that the permeate flow decreased as the salt content increased within the feed side [1,50,51]. Furthermore, the figure revealed that the reduction was just marginal [46]. The reason for this is because the addition of salt lowered the partial vapor pressure of water according to the modified Antoine equation, Equation (32), and therefore the driving force. This may also be impacted by a reduction in the convective heat transfer coefficient as substance concentration increases [6,52,53].

Figure 8 depicts the feed Reynolds number as a function of feedwater flow variation for pure water, brackish water, and seawater. The highest flow rate causes the Reynolds number to peak, which is explained by the high flow velocity.

**Figure 9:**
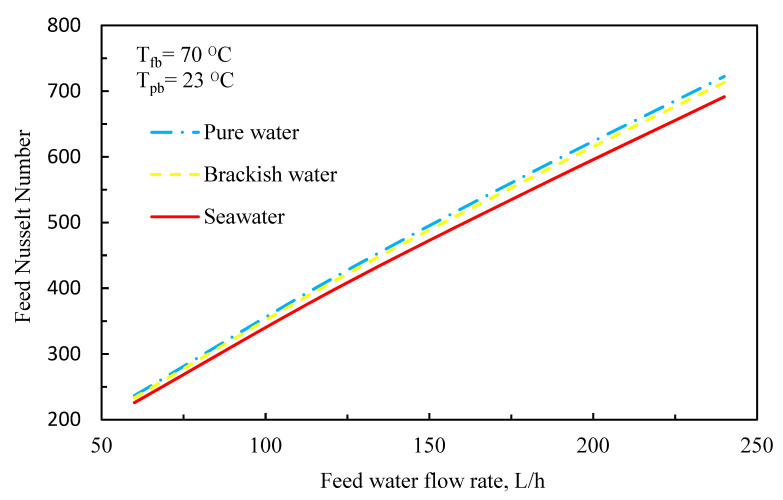
Feed Nusselt number versus feed water flow rate (*T_fb_* = 70 °C, *T_pb_* = 23 °C).

Also, the average Nusselt number increases by increasing the Reynolds number due to an increase in convection heat transfer over the membrane surface (Figure 9).

**Figure 10:**
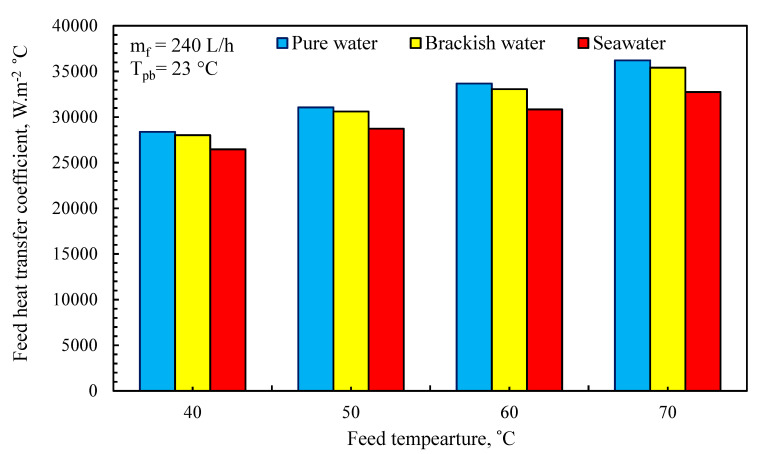
Effect of feed temperature on heat transfer coefficient (*m_f_* = 240 L/min, *T_pb_* = 23 °C).

The effect of feed water temperature on the numerical feed convection heat transfer coefficient is depicted in Figure 10. The presence of turbulent flow causes the peak values of the convection heat transfer coefficient to occur at high temperatures. The pure water feed convection heat transfer coefficient is 5% greater than that of saltwater. This significant increase in heat transfer coefficients is accomplished by increasing the feed temperature, which inhibits the formation of boundary layers and therefore reduces the thermal polarization influence.

**Figure 11:**
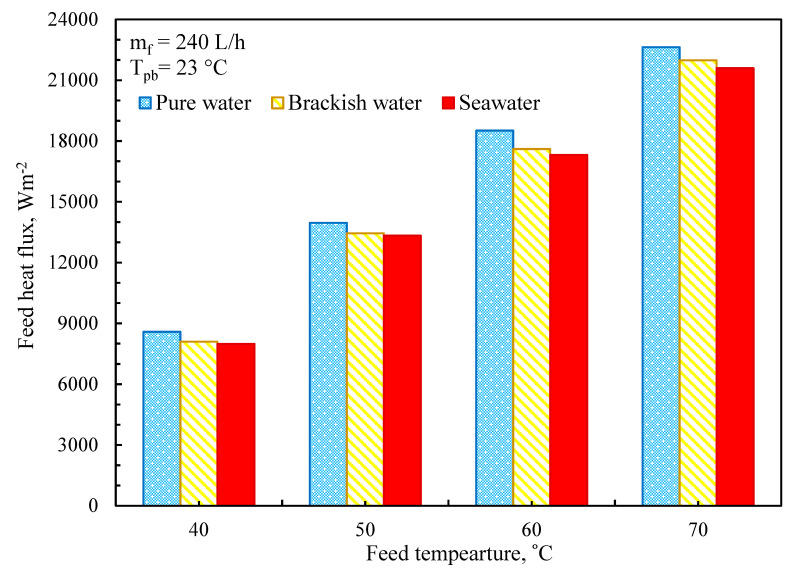
Effect of feed temperature on feed heat flux (*m_f_* = 240 L/min, *T_pb_* = 23 °C).

The significance of each heat transfer mechanism is resolved and is taken into account in terms of percentages when compared to total heat transfer rates. The percentage of feed heat transfer flux and percentage were used to describe the effect of mass transfer on heat transfer rates. The maximum percentages of feed heat flow at 40 and 70 °C, as shown in Figure 11, were 3.1 and 8.2 percent, respectively. Because of greater mass fluxes, feed heat flow increases with feed temperature. In Figure 10, the heat transfer coefficients are minimally influenced for feed heat transfer when the heat transfer coefficients grow, raising feed heat transfer and feed heat transfer within the same sections.

**Figure 12:**
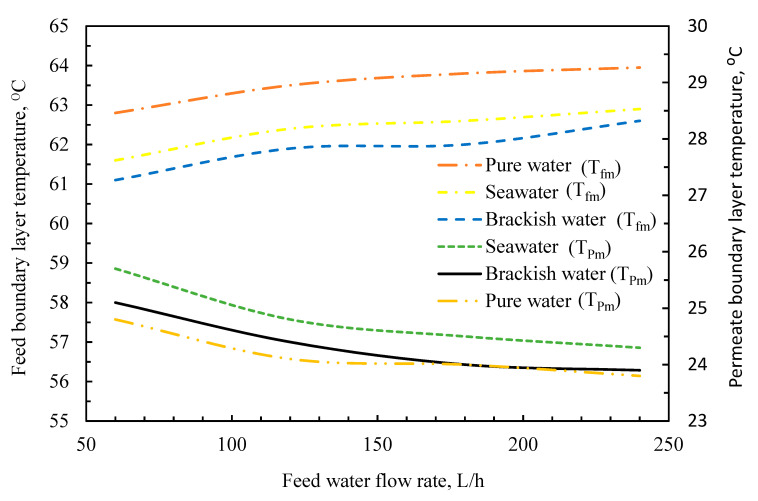
Feed membrane surface temperature versus feedwater flow rate.

Figure 12 depicts the average temperature of the membrane boundary layers on the membrane surface at various flow speeds. Within the picture, the temperature for seawater at the maximum point of the feed–membrane interface is around 57.5 °C, which is typically conventional because when the feed density is large, the temperature on the feed–membrane interface drops due to heat transfer processes. The permeate–membrane interface behaves similarly. In the same context, the graph indicates how close the amount of temperature dips in the feed facet and gains in the permeate facet is.

### 3.4. Temperature Polarization Effect

**Figure 13:**
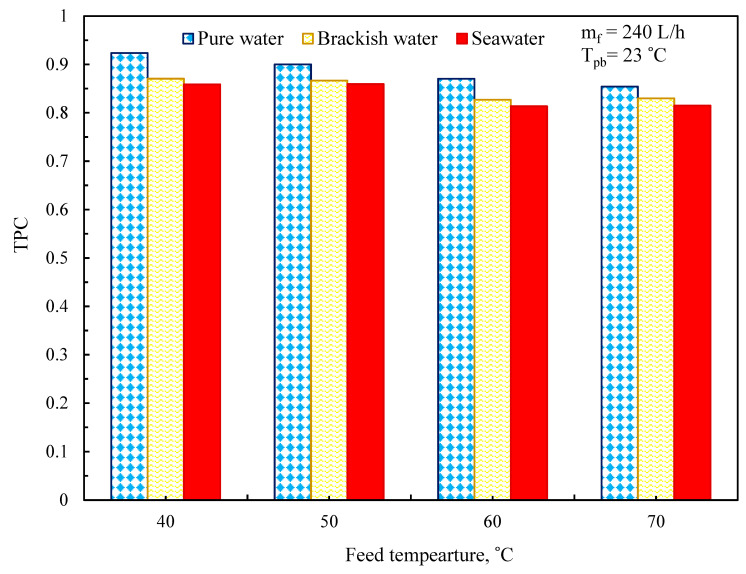
Influence of feed temperature on the temperature polarization factor.

TPC against feed temperature is plotted for NaCl solutions at a permeate temperature of 23 °C (Figure 13). TPC decreases with increasing feed temperature, which might be a well-recognized tendency in many membrane distillation processes [1,3,6,54]. This tendency might be explained by the fact that rising temperatures result in an increase in the energy consumed by water evaporation at higher temperatures. As a result, the temperature polarization influence is much more substantial, or, to put it another way, the temperature polarization coefficient (TPC) is smaller.

Figure 13 depicts the influence of feed temperature (*T_bf_*) on polarization coefficients (TPC). In general, temperature polarization coefficients decreased with *T_bf_* [1,20,36,44]. At greater temperatures, vaporization consumes more energy, which might explain this truth. Even while membrane factors impacting the MD process aren’t outside the scope of this study, a significant drop in temperature polarization influence has been seen due to increases in salt content and feed temperature. Based on the results, it is possible to conclude that in the case of low and high feed concentrations (brackish water TDS = 5 g/L, saltwater TDS = 35 g/L), TPC is less than 1% throughout the whole feed temperature range. In this case, TPC may be regarded as a good indication of the loss of a driving force as a consequence of temperature polarization, whereas this difference will increase with a rise in feed temperature from 40 to 70 °C, where the variance between TPC reached more than 10%.

**Figure 14:**
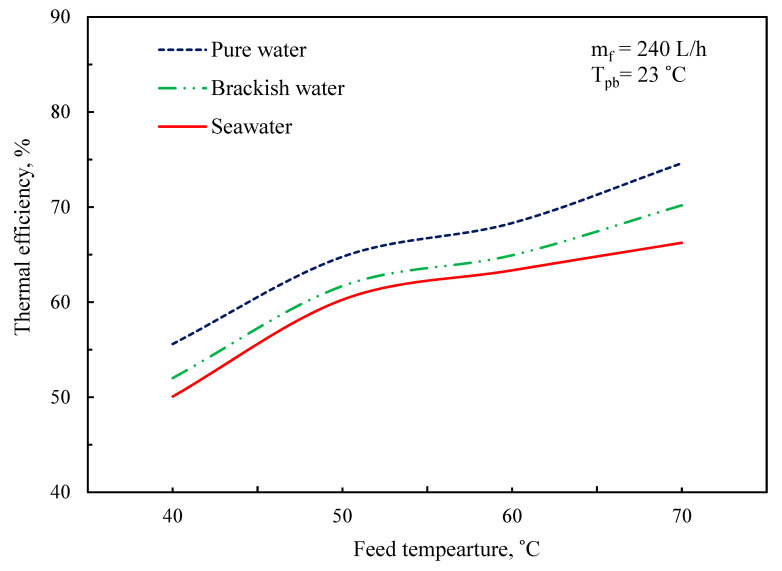
Influence of feed temperature on the thermal efficiency.

The impact of feed temperature on thermal efficiency for pure water, brackish water, and seawater is shown in Figure 14, which was defined previously in Equation (33). The relationship between thermal efficiency and temperature conditions is depicted in Figure 6 together with the feed side heat transfer coefficient. Because the input temperature rises from 40 to 70 °C for clean water, thermal efficiency rises from 55% to 72%. This observation is consistent with the observed trend for transmembrane permeate flow (Figure 7). The increased transmembrane flow suggests a larger proportion of heat carried through the membrane by convection, which rises the system’s thermal efficacy. The heat transfer factor, which measures the efficacy of heat transmission from the bulk to the membrane exterior, has a similar pattern. As the feed flow rises, the barrier to mass and heat transmission falls dramatically. As a result, heat transmission from the majority to the membrane surface becomes much more effective, and a greater heat transfer factor is obtained.

### 3.5. Thermal Performance of MD System

In MD setups, another element is considered when assessing the thermal performance of the MD system, specifically the specific thermal energy consumption (STEC), and the thermal energy provided, calculated as [55,56]:

(37) $$Q_{HR}=m_{f}\times C_{p}\times \left(T_{fi}-T_{fo}\right)$$

where $m_{f}$ denotes the feedwater flow rate (kg/h), $C_{p}$ the feedwater heat capacity (kWh/kg °C), $T_{fi}$ the feedwater inlet temperature (°C), and *T* the feedwater exit temperature (°C). The fundamental goal of the MD operation is to produce a large amount of freshwater while using as little energy as feasible. The required energy was calculated using the STEC (kWh/m^3^), which is defined as the amount of external heat required to create a quantity of freshwater, as stated by the following relationship between the energy efficiency ratio QHR and the permeate flow (J) [55].

(38) $$\mathrm{STEC}=Q_{HR}/J$$

**Figure 15:**
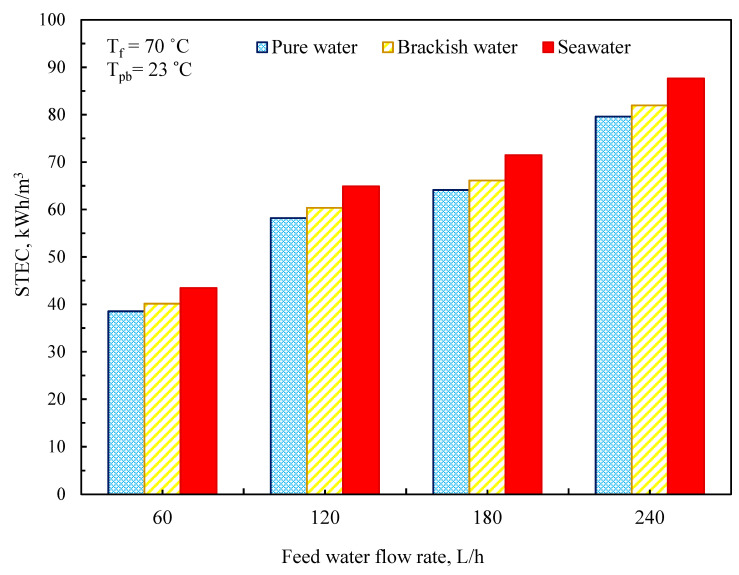
Specific heat energy consumption at a different feedwater flow rate.

The STEC values for varied input water flow rates and constant temperature at 70 °C are shown in Figure 15. The current study’s findings demonstrate that reducing the feedwater flow rate results in a long residence period and, as a result, a poor STEC. The STEC value of the DCMD module was found to be 39, 40, and 45 kWh/m^3^ for pure water, brackish water, and saltwater, respectively, at a feedwater flow rate of 60 L/h and a feed temperature of 70 °C, and rose to 80, 82, and 88 kWh/m^3^ when the feedwater flow was raised to 240 L/h.

## 4. Conclusions

The mass transport mechanisms of water vapor were examined in order to determine the primary membrane mass transference route of the MD process with clean water as a feed. According to the current study, the water vapor transference route is governed by the Knudsen molecular diffusion alteration mechanism, and its model was able to predict the investigational fluxes under operating circumstances. The mass transference factor of the tubular membrane computed using the Knudsen molecular transition model and found to be in close accord with the relevant experimental outcomes. Furthermore, it was discovered that, besides several membrane properties, the mass transference factor ($K_{m}$) is significantly temperature sensitive. The results of the mass transport resistances show that the operational parameters had an effect on the resistances, with the membrane transport resistance being the primary resistance influencing the evaporation flow, whereas the feed and permeate boundary film oppositions were significantly lower than that of the membrane resistance.

## Figures and Tables

**Table 1:** Thermal conductivity of some materials or gas involved in MD.

T	Polyvinylidene Fluoride	Polytetrafluoroethylene	Polypropylene	Air	Water Vapor
(K)	(W/m⋅K)				
296	0.17–0.19	0.25–0.27	0.11–0.16	0.026	0.022
348	0.21	0.29	0.20	0.03	0.022

**Table 2:** Correlation equations of the pure water and saline water.

Characteristic	Correlation	Conditions and Unit
Heat capacities of water [37]	*C_P_,_b_* = 1000(6.18507 − 0.0159(*T* + 273.15) + 3.99 × 10^5^(*T* + 273.15)^2^ − 3.06 × 10^−^^8^(*T* + 273.15)^3^)	16.85 °C < *T* < 96.85 °C, J/(kg °C)
Heat capacities of saline water [28]	*Cp_sw_* = 5.328 − 9.76 × 10^−2^*S* + 4.04×10^−4^*S*^2^ + (−6.913 × 10^−3^ + 7.351 × 10^−4^*S* 3.15 × 10^−6^*S*^2^)*T* + (9.6 × 10^−6^ − 1.927 × 10^−6^*S* + 8.23 × 10^−9^*S*^2^)*T*^2^ + (2.5 × 10^−9^ + 1.666 × 10^−9^*S* − 7.125 × 10^−12^*S*^2^)*T*^3^	273.15 K < *T* < 453.15 K; 0 < S < 180 g/kg, kJ/(kg k)
Latent heat of water vaporization [29,30]	∆*H_v_* = 2024.3 + 1.75535*T*	5 °C < *T* < 200 °C, J/(kg °C)
Density of liquid water [37]	*ρ_w_* = 1000(0.819 + 1.49 × 10^−3^(*T* + 273.15) − 2.9975 × 10^−6^(*T* + 273.15)^2^)	16.85 °C < *T* < 96.85 °C, kg/(m^3^)
Density of saline water [37]	$\rho_{s}=\frac{100}{\left(W_{Nacl}/2170\right)+\left(\left(100-W_{Nacl}\right)/\rho_{w}\right)}$	kg/(m^3^)
Viscosity of water vapor [37]	$\mu_{v}=-2.91\times 10^{-6}+4\times 10^{-8}\left(T_{m}+273.15\right)$	16.85 °C < *T* < 96.85 °C, kg/(m s)
Viscosity of liquid water [38]	$\mu_{w}=4.2844\times 10^{-5}+{\left(0.157{\left(T+64.993\right)}^{2}-91.296\right)}^{-1}$	0 °C ≤ *T* ≤ 180 °C, kg/(m s)
Viscosity of saline water [38,40]	$\mu_{sw}=1+AS+BS^{2}$ $A=1.474\times 10^{-3}+1.5\times 10^{-6}T-3.927\times 10^{-8}T^{2}$ $B=1.0734\times 10^{-5}-8.5\times 10^{-8}T+2.23\times 10^{-10}T^{2}$	10 °C < *T* < 180 °C; 0 < *S* < 150 g/kg, kg/(m s)
Thermal conductivity of liquid water [37]	$k_{b}=-0.465288+5.75172\times 10^{-3}\left(T_{m}+273.15\right)-7.1843\times 10^{-6}{\left(T_{m}+273.15\right)}^{2}$	20 °C < *T* < 100 °C, W/(m·°C)
Thermal conductivity of saline water [40]	$log_{10}\left(k_{sw}\right)=log_{10}\left(240+0.0002S\right)+0.434\left[2.3-\frac{343.5+0.037S}{T+273.15}\right]{\left[1-\frac{T+273.15}{647+0.03S}\right]}^{0.333}$	0 °C < *T* < 180 °C; 0 < *S* < 160 g/kg, W/(m·°C)

**Table 3:** Membrane specifications and operating conditions used in the simulations.

Parameter	Values
Thickness of membrane, δ	600 [μm]
Porosity of membrane, ε	51%
Pore size of membrane	0.72 [μm]
Tortuosity of membrane, *T*	1.96
Thermal conductivity of membrane, Km	0.27 [W/mK]
Feed Reynolds number, *Re_f_*	2500 to 15,000
Permeate Reynolds number, *Re_p_*	332
Concentration at feed inlet, *c_f_*, in	5000 and 35,000 [ppm]
Inlet feed temperature, *Tf*, in	40, 50, 60 and 70 °C
Inlet permeate temperature, *T_p_*, in	23 °C

**Table 4:** Specifications of the measuring devices and values of uncertainty.

Device	Accuracy	Range	Standard Uncertainty
Thermocouple	0.15 °C	0–150 °C	0.086 °C
Rotameter	0.1 L/min	8 L/min	0.057 L/min
TDS meter	5 ppm	0–50,000 ppm	2.89 ppm
Balance	0.5 g	1 to 25,000 g	0.289 g

**Table 5:** Influence of the Knudsen number on a mass transfer through a porous medium.

Driving Force	$K_{n}\;<\;0.01$	$0.01\;<\;K_{n}\;<\;1$	$K_{n}\;>\;1$
Gas Mixture ΔP=0, $\Delta p_{A}\neq 0,or\;\Delta y_{A}\neq 0$	M	M–K transition	K

**Table 6:** Comparison between the theoretical and experimental value of the mass transfer coefficient.

Method	$K_{m}\times 10^{-3}\left(kg/m^{2}\cdot h\cdot Pa\right)$
Knudsen diffusion	0.75
Molecular diffusion	0.66
K-M transition	0.35
Experimental	0.39

**Table 7:** The influence of temperatures on mass transport resistances.

$T_{f}\;\left({}^{\circ}C\right)$	$R_{f}{}_{\mathrm{Exp.}}$	$R_{f}{}_{Theor.}$	$R_{m}{}_{Exp.}$	$R_{m}{}_{Theor.}$	$R_{p}{}_{Exp.}$	$R_{p}{}_{Theor.}$
40	210.36	225.12	2821.54	3019.55	108.09	115.68
50	70.48	81.42	2534.76	2928.51	35.03	40.48
60	73.89	76.39	2752.95	2845.95	37.48	38.74
70	690.24	698.04	2681.88	2712.18	377.40	381.66

## Data Availability

The data that support the findings of this study are available from the corresponding author upon reasonable request.

## List of Symbols

$a_{w}$	Water activity
$A_{m}$	Area of the membrane $\left({\mathrm{cm}}^{2}\right)$
$A_{T}$	Area of Tank $\;\left({\mathrm{cm}}^{2}\right)$
$c_{p}$	Heat capacity (J/kg·K)
CFV	Cross flow velocity (m/s)
C	Molar concentration of the solution (mol/L)
$C_{fb}$	Molar concentration at feed temperature
$C_{fm}$	Molar concentration at the membrane surface
$d_{p}$	Membrane pore size diameter (µm)
$d_{h}$	Hydraulic diameter (m)
$D_{AB}$	Diffusivity of solute (m^2^/s)
$D_{wa}$	Diffusivity of water vapor-air mixture (m^2^/s)
F	Water feed flow rate (mL/min)
$\Delta H_{v}$	Latent Heat of vaporization (kJ/kg)
*h*	Heat transfer coefficient $\left(W/m^{2}K\right)$
J	Mass vapor flux (kg/m^2^·h)
$J_{K}$	Knudsen diffusion flux (kg/m^2^·h)
$J_{M}$	Molecular diffusion flux (kg/m^2^·h)
$J_{P}$	Poiseuille flow flux
k	Thermal conductivity at the polarization layers (W/m·K)
k	Thermal conductivity (W/m·K)
$K_{m}$	Mass transfer coefficient (kg/m^2^·h·Pa)
$L_{m}$	Membrane length (mm)
*M*	Molality of NaCl in NaCl solution (mol/kg)
$\overset{•}{m}$	Mass flow rate (kg/s)
$M_{w}$	Molecular weight of water (kg/kmol)
P	Pressure (Pa)
Q	Heat flux (W/m^2^)
*RH*	Relative humidity
*R*	Resistance at feed boundary layer $\;\left(\mathrm{Pa}\cdot m^{2}h/\mathrm{kg}\right)$
$T_{m}$	Mean temperature (°C, K)
*T*	Temperature (°C, K)
t	Time (s)
V	Volume of the tank $\left({\mathrm{cm}}^{3}\right)$
v	Fluid velocity (m/s)
Dimensionless numbers	
$K_{n}$	Knudsen number
*Re*	Reynolds number
*Sc*	Schmidt number
*Sh*	Sherwood number
*CP*	Concentration polarization coefficient (CP)
Greek letters	
*α*	Reynolds number exponent
*β*	Schmidt number exponent
*ρ*	Fluid density (kg/m^3^)
*μ*	Fluid viscosity (Pa·s)
δ	Membrane thickness (m)
*τ*	Membrane tortuosity
*ε*	Membrane porosity
λ	Mean free path (m)
Subscripts	
*b*	Bulk
*c*	Conduction
*g*	Gas
*Exp.*	Experimental
*f*	Feed
*m*	Membrane
*MD*	Membrane Distillation
*M*	Molecular diffusion
*K*	Knudsen diffusion
*K-M*	Knudsen-Molecular transition diffusion
*P*	Permeate
*s*	Salt
*v*	Vaporization
1	Membrane location at feed side
2	Membrane location at permeate side
